# Expressed Sequence Tags Analysis and Design of Simple Sequence Repeats Markers from a Full-Length cDNA Library in *Perilla frutescens* (L.)

**DOI:** 10.1155/2015/679548

**Published:** 2015-11-19

**Authors:** Eun Soo Seong, Ji Hye Yoo, Jae Hoo Choi, Chang Heum Kim, Mi Ran Jeon, Byeong Ju Kang, Jae Geun Lee, Seon Kang Choi, Bimal Kumar Ghimire, Chang Yeon Yu

**Affiliations:** ^1^Bioherb Research Institute, Kangwon National University, Chuncheon 200-701, Republic of Korea; ^2^Department of Bioconvergence Science and Technology, College of Agriculture and Life Sciences, Kangwon National University, Chuncheon 200-701, Republic of Korea; ^3^Hwajin Cosmetics, Hongcheon 250-807, Republic of Korea; ^4^Department of Agricultural Life Sciences, Kangwon National University, Chuncheon 200-701, Republic of Korea; ^5^Department of Applied Bioscience, Konkuk University, Seoul 143-701, Republic of Korea

## Abstract

*Perilla frutescens* is valuable as a medicinal plant as well as a natural medicine and functional food. However, comparative genomics analyses of *P. frutescens* are limited due to a lack of gene annotations and characterization. A full-length cDNA library from *P. frutescens* leaves was constructed to identify functional gene clusters and probable EST-SSR markers via analysis of 1,056 expressed sequence tags. Unigene assembly was performed using basic local alignment search tool (BLAST) homology searches and annotated Gene Ontology (GO). A total of 18 simple sequence repeats (SSRs) were designed as primer pairs. This study is the first to report comparative genomics and EST-SSR markers from *P. frutescens* will help gene discovery and provide an important source for functional genomics and molecular genetic research in this interesting medicinal plant.

## 1. Introduction

*Perilla frutescens* (L.) is a self-compatible annual herb known as the beefsteak mint plant. It is cultivated in East Asian countries, including Japan, China, and Korea, and is an economical crop in the medicinal herb family, Lamiaceae [1]. Its seeds can be processed into foods and nutritional edible oils, and its leaves can be utilized as a traditional medicinal herb or flavor for vegetables [2, 3]. Perilla oil contains abundant polyunsaturated fatty acids (PUFAs), including linolenic (56.8%) and linoleic (17.6%) acids, which are used in salad oils or cooking [4, 5]. The flavor and odor of perilla are caused by the essential oils of monoterpenoids and sesquiterpenoids, including terpenoids, and they are commercially used as a natural fragrance or for flavoring [6]. The perilla leaf is composed of a number of chemical variants of the volatile essential oil classified as PA-type (mainly in perillaldehyde), EK-type (elsholtziaketone), PK-type (perilla ketone), PL-type (perillene), PP-type (phenylpropanoids), and PT-type (piperitenone) [7]. Perilla has been described as an important pharmaceutical with anti-inflammatory, anti-allergic, and broad antioxidant functions [8, 9].

Expressed sequence tags (ESTs) are fragments of expressed genes occurring from single-pass sequencing of cDNA libraries [10]. EST databases are sources of SSRs that can be developed as ortholog-specific EST-SSR markers and are dependent on genotype applications in many plant species [11–18]. As a molecular tool, EST-SSRs are highly important for studies on genetic populations [19]. They can identify functional markers in the open reading frames (ORFs) or 5′- or 3′-untranslated regions (UTRs) as well as exerting a phenotypic effect [20]. One advantage of the EST-SSR is that it is more transferable across closely related genera compared with unknown SSRs in the UTRs or noncoding sequences. Therefore, EST-SSRs are easy to understand for studying polymorphisms and genetic diversity [21, 22]. EST-derived SSRs have been reported in various plant species, including* Arabidopsis thaliana*, cacao, and sugarcane [23–25]. EST-SSRs also provide a new source for genetic and evolutionary studies based on homology searches of putative SSR functions [26].

In this study, we developed a full-length enriched cDNA library from* P. frutescens* leaves. EST sequence analysis allowed for genome annotation and gene ontologies and the identification of EST-SSR markers for genomic tool development in this less-well-studied medicinal plant species. These results provide useful and multipurpose data for further studies on* P. frutescens*.

## 2. Materials and Methods

### 2.1. Plant Materials

Seeds of* P. frutescens* were obtained after harvest in attached farm of Kangwon National University (Republic of Korea) during each year of collected accessions and grown on pot supplemented with commercial soil (GFC, Hongseong, Republic of Korea) in a greenhouse for a photoperiod of 16-hour light/8-hour dark at 25°C under well-water conditions. Leaves were sampled for RNA isolation.

### 2.2. RNA Extraction, cDNA Library Construction, Plasmid DNA Extraction, and Sequencing

Leaves were ground with a pestle in the presence of liquid nitrogen and ground tissue was used to RNA isolation using the Trizol method [27]. Total RNA was stored at −80°C until use. The full-length cDNA library was constructed using the Creator SMART cDNA Construction Kit (Clontech Laboratories, CA, USA). Concentrations of isolated RNAs were evaluated using a nanodrop spectrophotometer (Thermo Fisher Scientific, Wilmington, DE, USA) and then used for first-strand cDNA synthesis. Second-strand cDNA was purified by QIAquick (Qiagen, Venlo, Netherlands) and ligated or transformed into the pTripleEX Vector. Plasmid DNA extraction was processed using a Multiscreen Plasmid Extraction Kit (Millipore) and purified. The cDNA library was amplified and using GeneAmp PCR System 9700–384 (Applied Biosystems, CA, USA) and DNA clones was sequenced with single-pass sequencing from the 5′-ends of the cDNA.

### 2.3. Assembly Annotation

Because genome and gene information were unavailable, assembly was performed without clustering. In the pretreatment process, PHRED was used to transfer peak information into the quality file and trim low-quality bases. Vector sequences were trimmed using Cross_match (http://www.macvector.com/Assembler/trimmingwithcrossmatch.html). Chimeric clones, polyA-tails, and sequences less than 100 bp were removed with Seqclean. Assembly was performed with CAP3 software. Contigs were manually checked and, together with singlet reads, compiled to generate a final unigene file. Finally, 412 unigene sequences were obtained from 1,000 ESTs, which were composed of 69 contigs and 343 singletons.

Unigenes were searched against the NCBI nonredundant nucleotide (NT) and protein (NR) databases (http://www.ncbi.nlm.nih.gov/), the Uniprot_sprot database (http://www.ebi.ac.uk/), and BLAST2GO (https://www.blast2go.com/) for functional annotation using the BLAST alignment tool. A sequence was considered as a significant match when the BLAST probability value (E-value) was less than 1e-5, and the match with the most significant E-value was recognized as the best annotation. A BLASTx search was also conducted against the UniProtKB/Swissprot database (http://www.ebi.ac.uk/) using default parameters. Unigenes were further annotated with GO terms (http://geneontology.org/).

### 2.4. Primer Design for EST-SSR Markers

A total of 1,000 ESTs of 1,056 samples obtained from a cDNA library were detected and analyzed by TRF version 4.07b online software (http://tandem.bu.edu/trf/trf.html). SSR sequences were then obtained. SSRs that fit the following criteria were considered for primer design: a minimum length of 18 bp with minimum repetitions for di-, tri-, tetra-, penta-, and hexa-4 and 4, respectively. Primers were designed using Primer 3 (http://www.premierbiosoft.com/primerdesign/) according to the following core criteria: a primer length ranging from 18 bp to 22 bp, with 20 bp as the optimum; product size ranging from 100 bp to 400 bp; melting temperature between 50°C and 62°C, with 60°C as the optimum; and GC content between 40% and 60%, with avoidance of mismatch, hairpin structures, and primer dimers that can cause nonspecific amplification.

## 3. Results

### 3.1. cDNA Library Quality Check and Reads Assembly

**Figure 1:**
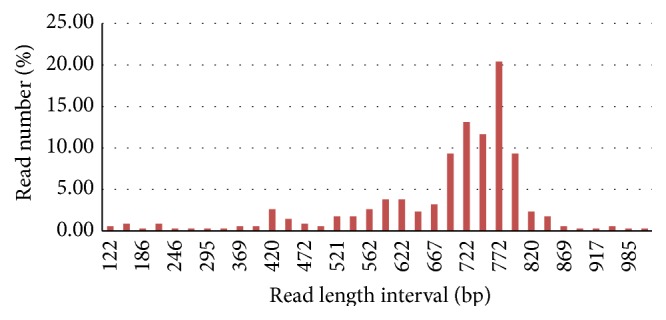
Reads length representation in EST (expressed sequence tags) sequencing of* Perilla frutescens*. Range of read length was indicated from 121 bps to 1051 bps.

A full-length cDNA library was constructed from a mixture of* P. frutescens* samples. Library quality was evaluated after sequencing 96 randomly selected clones. On average, the insert size was greater than 1.2 kb. Forty-nine clones (51.04%) yielded sequencing reads above 700 bp; 11 clones (11.45%) were less than 500 bp. After confirming clone quality, a mass-scale sequencing approach was used. Construction of the full-length cDNA library was produced from* P. frutescens*. A total of 1,000 randomly selected clones from the cDNA library were subjected to single-orientation sequencing from the 5′-end using an ABI3730xl Platform (BGI). Read lengths ranged from 420 bp to 844 bp, with an average of 632 bp (Figure 1).

### 3.2. GC Content by Assembly of cDNA Reads

**Figure 2:**
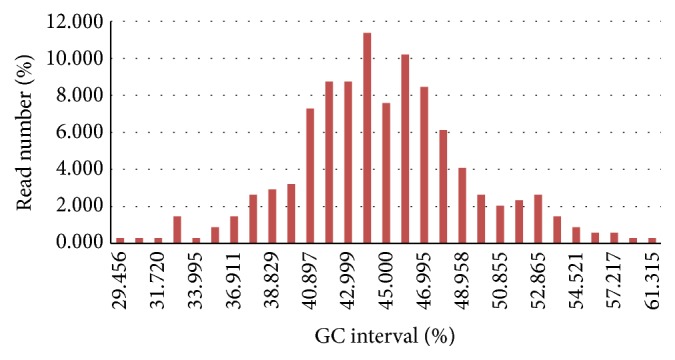
GC content division of unigenes. GC content of unigenes changed from 29.45% to 61.32%.

One thousand EST reads were obtained by trimming vector contaminants with Crossmatch and eliminating chimeric clones and short sequences (less than 100 bp). EST reads were then assembled by PHRAP and CAPS software [28, 29]. Results from the CAP3 assembly indicated that the GC content of unigenes varied from 29.46% to 61.32%. Ninety-one percent of the unigenes exhibited GC content between 37.93% and 52.87% (Figure 2).

### 3.3. Sequence Annotation

Annotation of the EST library was achieved through BLAST (Table 1). The NCBI nonredundant nucleotide (NT) (BLASTn) database resulted in 312 (90.96%) unigenes, whereas the protein (NR) database (BLASTx) produced 322 (93.88%) annotations. Uniprot/Swissprot (BLASTx) databases revealed the annotation of 317 (92.42%) unigenes. Moreover, the annotation data from COG (BLASTx) classification revealed 111 (32.36%) unigenes. Results from the NR database were determined to match that of the sequence homology with two species,* Sesamum indicum* (185 genes, 57.45) and* Erythranthe guttata* (78 genes, 24.22%). The remaining genes exhibited low levels (less than 1.86%) of sequence homology (Table 2).

### 3.4. Classification of Annotated Genes by GO Analysis

**Figure 3:**
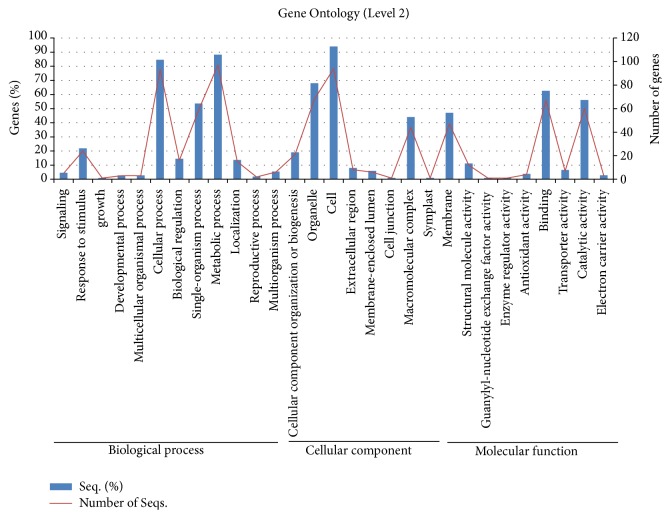
Classification of unigenes by Gene Ontology (GO) analysis. Three major clusters were displayed with annotated genes at hierarchy level 2 of GO analysis.

Gene Ontology (GO) distribution using hierarchy level 2 of the GO program resulted in three major clusters: biological process, cellular component, and molecular function (Figure 3). First, the biological process group was separated into 13 subclasses: signaling (5 genes), response to stimulus (24 genes), growth (1 gene), developmental process (3 genes), multicellular organismal process (3 genes), cellular process (93 genes), biological regulation (16 genes), single-organism process (59 genes), metabolic process (97 genes), localization (15 genes), reproductive process (2 genes), multiorganism process (6 genes), and cellular component organization or biogenesis (21 genes). Organelle (68 genes), cell (94 genes), extracellular region (8 genes), membrane-enclosed lumen (6 genes), cell junction (1 gene), macromolecular complex (44 genes), symplast (1 gene), and membrane (47 genes) genes were distributed from the cellular component cluster. The major components of the molecular function subset consisted of binding (67 genes) and catalytic activity (60 genes) genes.

### 3.5. EST-SSR Traits in* P. frutescens*

A total of 343 unigene sequences were investigated. SSR sequences were obtained using TRF version 4.07b online software. Eighteen EST-SSR sequences were selected and analyzed following functional annotation (Table 3). Primer pairs were designed using the Primer 3 program. Expected product sizes ranged from 191 bp to 773 bp. In the future, we will perform additional classification studies through gene functions of* P. frutescens* using these EST-SSR primers.

## 4. Discussion

The major outcomes of this study were the construction of a full-length cDNA library from the important* P. frutescens* L-type (with limonene component) and the preliminary 1,000 ESTs identified (average 632 bp in length). Genome segment quality was affected by many factors. GC content analysis revealed a distribution between 29.46% and 61.32%. Earlier study showed that thirty to fifty percent of GC content influenced genome sequence quality in* Medicago truncatula* and* Lotus japonicas* [30]. GC content increment was related to the ratio of segments with matching EST data [31], consistent with that from the human genome [32].

Gene Ontology (GO) was utilized to obtain functional information and descriptions of gene products by studying domain-specific ontologies [33]. Annotation results consisted of biological data related to stress response genes, which were classified functionally using the GO hierarchy [34]. The corresponding classifications were processed to obtain additional information on the putative functionality for the subject accession number of pepper EST data from the GO databases [35]. GO “biological process” and “molecular function,” generated by level 3, were annotated and associated with the number of sequences from each term, which were normalized by labeling with a GO term [36].

We also established 18 EST-SSR primers from the full-length cDNA library of* P. frutescens*. In* Vitis vinifera*,* Artemisia tridentate*,* Panax ginseng*, and* S. miltiorrhiza*, the EST-SSR motifs were generally di- and trinucleotide repeats [37–40]. However, this study revealed various penta-, hexa-, dodeca-, and tetradecanucleotide repeat motifs. This finding is in agreement with that for* Scutellaria baicalensis*, which contains penta- and hexanucleotide repeats [41]. Differences in repeat type may be attributed to the degree of the SSR search criteria for the EST database in various plant species. The development of EST-SSR markers has many advantages compared with other molecular markers and can be used to study genetic diversity, evolution, comparative genomics, and gene-based associations.

Construction of a full-length cDNA library is significant for comparative genomics, genome sequence validation, and design of EST-SSR primers that display entire transcription units rather than partial gene sequences [42]. One benefit of constructing a full-length cDNA library is that it allowed us to conduct proper gene modeling while comparing other cDNA sequences in* P. frutescens*. The full-length cDNA sequences will be useful for annotation of the plant genome. Another advantage of EST sequencing is the increased ratio of unigenes with definitive GO categories compared with other libraries. The library built by this method included a high proportion of full-length cDNAs [42], allowing us to have a database of this library available for* P. frutescens* genomics studies.

This full-length cDNA library provides a wealth of knowledge about the unique EST sequences available for the* P. frutescens* genome and, particularly, about the addition of 5′-end sequences that are more unique and valuable for gene identification. These EST tags will be useful for functional gene annotation, analysis of splice site variations, and gene homologies as additional whole-genome sequences become available in* P. frutescens*.

## 5. Conclusions

*Perilla frutescens* is valuable as a medicinal plant as well as a natural medicine and functional food. However, comparative genomics analyses of* P. frutescens* are limited due to a lack of gene annotations and characterization. A full-length cDNA library from* P. frutescens* leaves was constructed to identify functional gene clusters and probable EST-SSR markers through 1,056 examples of expressed sequence tag (EST) sequencing data. Unigene assembly was performed using basic local alignment search tool (BLAST) homology searches and annotated Gene Ontology (GO). A total of 18 simple sequence repeats (SSRs) were designed as primer pairs. This study is the first to report comparative genomics and EST-SSR markers from* P. frutescens* to ease gene discovery and provide an important source for functional genomics and molecular genetic research in this interesting medicinal plant.

## Figures and Tables

**Table 1:** Annotated unigenes from different databases by EST (expressed sequence tags) sequencing of *Perilla frutescens*.

Annotation DB (methods)	Hits	%	No hits	%
NT (BLASTn)	312	90.96%	31	9.04%
NR (BLASTx)	322	93.88%	21	6.12%
Uniprot + Swissprot (BLASTx)	317	92.42%	26	7.58%
COG (BLASTx)	111	32.36%	232	67.64%

**Table 2:** List of species containing sequence matches to *Perilla frutescens*.

Species (total: 38)	Genes (total: 322)
*Sesamum indicum*	185
*Erythranthe guttata*	78
*Salvia miltiorrhiza*	6
*Coffea canephora*	4
*Vitis vinifera*	4
*Nicotiana sylvestris*	4
*Genlisea aurea*	3
*Prunus persica*	2
*Nicotiana tomentosiformis*	2
*Ricinus communis*	2
*Brassica napus*	2
*Gossypium arboreum*	2
*Phoenix dactylifera*	2
*Perilla frutescens*	2

*Prunus mume*	1
*Medicago truncatula*	1
*Malus domestica*	1
*Solanum tuberosum*	1
*Schiedea haleakalensis*	1
*Mentha *×* piperita*	1
*Ajuga reptans*	1
*Codiaeum variegatum*	1
*Scutellaria baicalensis*	1
*Nicotiana tabacum*	1
*Morus notabilis*	1
*Citrus sinensis*	1
*Eucalyptus grandis*	1
*Eutrema salsugineum*	1
*Citrus clementina*	1
*Elaeis guineensis*	1
*Tarenaya hassleriana*	1
*Miscanthus sinensis*	1
*Arabidopsis thaliana*	1
*Arachis diogoi*	1
*Glycine max*	1
*Populus trichocarpa*	1
*Lolium perenne*	1
*Jatropha curcas*	1

**Table 3:** EST-SSR primer pairs produced in EST (expressed sequence tags) sequencing database of *Perilla frutescens*.

Number	Unigene ID	Repeat motifs	Left primer sequence	Tm.	Right primer sequence	Tm.	Product size (bp)	Annotation (NR DB)
1	Contig 17	ATCAT(8)	GAGAGTATAAACAAATCCAAAACAGC	58.795	AGCCGGTATATCCAATTCCC	60.006	562	PREDICTED: protein CURVATURE THYLAKOID 1B, chloroplastic [Sesamum indicum]
2	Contig 67	A(29)	AGCAACTGCGGGTAGCTAGA	60.176	CAATCCGACCACAGTTGATG	59.96	172	PREDICTED: photosystem I subunit O [Sesamum indicum]
3	Perilla-1-1a_pTriplEx2-seq_C16	GA(16)	AGCGTACTGTTGAAAGCGTG	59.148	CAGCAAACGTGCTCGAATTA	60.014	247	PREDICTED: uncharacterized protein LOC105172991 isoform X2 [Sesamum indicum]
4	Perilla-1-1a_pTriplEx2-seq_E18	CTT(9)	GCCAATTTGAAGCTTTAGCC	58.969	GAATGTGAAGTGGGAACGCT	60.119	773	PREDICTED: GRF1-interacting factor 3 [Sesamum indicum]
5	Perilla-1-1a_pTriplEx2-seq_M02	AGAATG(4)	TGGAGCAAGTGAAGCAACAG	60.175	CCTTTTCAGTGAGGAGCCAG	59.982	191	
6	Perilla-1-2a_pTriplEx2-seq_A02	TTTTG(7)	AATGATGGGTGTGATGAGCA	59.925	AAAGAATTTGAAGGCGCAGA	59.96	401	PREDICTED: homeobox-leucine zipper protein HAT5-like [Sesamum indicum]
7	Perilla-1-3a_pTriplEx2-seq_B13	ATCAT(8)	GAGAGTATAAACAAATCCAAAACAGC	58.795	CGGTATATCCAATTCCCACG	60.031	559	hypothetical protein MIMGU_mgv1a015066mg [Erythranthe guttata]
8	Perilla-1-3a_pTriplEx2-seq_L05	CT(14)	CCCAAATTCACATCCACTGA	59.343	AACAACTGACATGGCCTTCC	59.973	185	PREDICTED: uncharacterized protein LOC105160440 [Sesamum indicum]
9	Perilla-1-3a_pTriplEx2-seq_L15	TC(15)	CAGTTTTAACTTCGCCTCGC	60.018	CACTCGCAAAAAGGGGTAAG	59.741	619	PREDICTED: annexin D5 [Sesamum indicum]
10	Perilla-2-1a_pTriplEx2-seq_A19	GGA(8)	GCTCCTCGCAGTAACTTTGG	60.015	TCATCTCTTGCTCTGTTTCCA	58.583	107	hypothetical protein MIMGU_mgv1a016040mg [Erythranthe guttata]
11	Perilla-2-2a_pTriplEx2-seq_A06	CT(12)	CATTGGCCTTAAACTTCGGA	60.067	ATAAATGTGGATTGGGGCAA	60.016	341	hypothetical protein MIMGU_mgv1a020048mg [Erythranthe guttata]
12	Perilla-2-2a_pTriplEx2-seq_C18	AG(14)	GGGGGATCATTTCCAGTCTT	60.133	GTGCCCACTGGTTCTTTGTT	60.012	404	hypothetical protein MIMGU_mgv1a012334mg [Erythranthe guttata]
13	Perilla-3-2a_pTriplEx2-seq_E14	GATGACGATGAT(2)	CTTTCCAACCCTCCGAATTT	60.291	CGACGCCTGTCTCATCTACA	60.008	522	NAC transcription factor 1 [Salvia miltiorrhiza]
14	Perilla-3-2a_pTriplEx2-seq_O22	GA(17)	GGGGATATGTTATGTTGCTTGTT	59.179	TCGCCGTACTTGATCCCTAC	60.096	514	PREDICTED: uncharacterized protein LOC105169169 isoform X1 [Sesamum indicum]
15	Perilla-3-3a_pTriplEx2-seq_B03	CT(16)	CGAGTGTGTTCGTATGGGTG	60.025	AACGCGTACGGAACAGAGAC	60.321	184	hypothetical protein MIMGU_mgv1a014836mg [Erythranthe guttata]
16	Perilla-3-3a_pTriplEx2-seq_B23	TCCTCTTCCTCTCC(2)	TAGTGTCGAAGCTCAATGGC	59.028	TGACCAGCATCAGCTTTCAC	59.992	662	PREDICTED: chloroplast stem-loop binding protein of 41 kDa a, chloroplastic [Sesamum indicum]
17	Perilla-3-3a_pTriplEx2-seq_F11	GAG(9)	GAAAGACTGGTTGGCTCTGG	59.844	ATCCAAAATTCGTCCTGTGC	59.939	381	hypothetical protein MIMGU_mgv1a011207mg [Erythranthe guttata]
18	Perilla-3-3a_pTriplEx2-seq_P03	GA(13)	AAAGCTGTTTGCCCTTGCTA	60.018	CTCAAATGGAGTCACGCAGA	59.984	284	hypothetical protein MIMGU_mgv1a016830mg [Erythranthe guttata]
